# Investigation of the Female Athlete Triad in Japanese Elite Wheelchair Basketball Players

**DOI:** 10.3390/medicina56010010

**Published:** 2019-12-27

**Authors:** Yukiyo Shimizu, Hirotaka Mutsuzaki, Kaori Tachibana, Kazushi Hotta, Yasuyoshi Wadano

**Affiliations:** 1 Department of Rehabilitation Medicine, University of Tsukuba Hospital, Tsukuba 305-8576, Japan; 2 Department of Rehabilitation Medicine, Ibaraki Prefectural University Hospital of Health Sciences, Ami 300-0331, Japan; 3 Department of Orthopaedic Surgery, Ibaraki Prefectural University of Health Sciences Hospital, Ami 300-0331, Japan; mutsuzaki@ipu.ac.jp; 4 Department of Physical Therapy, School of Healthcare, Ibaraki Prefectural University of Health Sciences, Ami 300-0394, Japan; tachibana@ipu.ac.jp; 5 Department of Occupational Therapy, School of Healthcare, Ibaraki Prefectural University of Health Sciences, Ami 300-0394, Japan; hotta@ipu.ac.jp; 6 Department of Orthopaedic Surgery, Miho Clinic, Miho 300-0415, Japan; y_wadano@k-kyojokai.or.jp

**Keywords:** female athlete triad, para-athlete, wheelchair basketball, low energy availability, bone mineral density

## Abstract

*Background and objectives:* Para-sports have become increasingly competitive, necessitating greater physical activity; secondary disorder prevention is therefore crucial. Among secondary disorders, the female athlete triad (FAT) is defined as low energy availability (EA), menstrual dysfunction, and low bone mineral density (BMD); although studied in able-bodied athletes, reports on female para-athletes are scarce. We retrospectively investigated the FAT in wheelchair basketball players in the Japanese national team. *Materials and Methods:* Thirteen female wheelchair basketball players (mean age: 28.9 ± 8.1 years) were enrolled. The medical history (underlying diseases, gynecological disorders, and stress fractures), athletic and sport-specific parameters (wheelchair basketball classification, and wheelchair usage conditions), hematological status (hemoglobin, iron, estradiol, progesterone, total P1NP, and TRACP-5b levels), nutritional status (total energy, protein, calcium, and iron intake), body composition (BMD and lean body mass (LBM)), and EA were assessed. *Results:* Two (15.4%) had pertinent gynecological histories and six (46.2%) had menstrual cycle disorders. Three (23.1%) experienced excessive menstrual flow and nine (69.2%) had menstrual pain. No stress fractures were reported. All laboratory data were within normal limits. Total energy and iron intakes based on age-specific requirements were 99.8% and 59.8%, respectively. Iron and hemoglobin levels correlated with menstrual flow (ρ = −0.63, *p* = 0.019 and ρ = −0.56, *p* = 0.046, respectively). The mean total BMD was 109.2%, and the mean EA (41.4 kcal/kg LBM) was lower than recommended levels. The leg BMD in spinal disorders was significantly lower than that in skeletal disorders (*p* = 0.003). The arm LBM was higher (150.6%) than that of age-matched controls. *Conclusion:* Among female wheelchair basketball players with FAT, the total BMD was comparable to that of age-matched controls; however, leg BMD in spinal disorders was significantly lower than that in skeletal disorders. Players with heavy menstrual flow had lower hemoglobin and iron levels. Further research is needed on the FAT to optimize health and sports performance among para-athletes.

## 1. Introduction

Adaptive sports have gained popularity; the increased competitiveness therefore necessitates better ways of preventing secondary disorders. The female athlete triad (FAT) is an important concept in female athlete performance and sports. It is defined as low energy availability (EA), menstrual dysfunction, and low bone mineral density (BMD) [1,2,3,4]. 

Low EA may cause menstrual disorders and low BMD. This concept has been studied in able-bodied athletes [1,3,4]. However, evidence in para-athletes is scarce, and this population may present with other problems [2]. There are many para-athletes who have spinal cord injuries, or use a wheelchair for daily locomotion. They have muscle atrophy in lower limbs and their arms work for handling the wheelchair. Wheelchair users with spinal cord injuries have an increased risk of osteoporosis due to the lack of skeletal loading [5]. BMD rapidly decreases after traumatic SCI and, furthermore, disorganization of trabecula bone architecture occurs owing to the decrease of the mechanical stress [6,7,8,9]. Moreover, athletes with skeletal disorders such as amputations require more energy owing to compensatory gait [10]. There are many amputees whose unaffected limbs are over loaded, and many players with skeletal disorders with multiple joint disorders, especially after high energetic accidents. They need more support regarding the FAT; however, few reports have focused on female para-athletes. Therefore, further research is needed on the characteristics of the FAT in para-athletes to adequately optimize their health and performance. The nature of the sports and related disorders should also be clearly understood. Wheelchair basketball is a major para-sport with >1000 players in Japan and >100,000 players worldwide [11]. Players may have skeletal or central nervous system diseases, and may be daily wheelchair users or non-users. Wheelchair basketball players are classified into eight categories (1.0, 1.5, 2.0, 2.5, 3.0, 3.5, 4.0, and 4.5) according to players’ trunk stability by the International Wheelchair Basketball Federation [12,13]. According to the classification, players perform differently in games. Athletes have different backgrounds in daily life or in games. Players with spinal cord injuries may have more risk of fractures, or high-pointer players might have more mechanical stress in the games. Therefore, different types of support in the FAT are necessary. We have studied factors associated with deep tissue injuries [14,15] and shoulder pain [16] in Japanese elite wheelchair basketball players. We identified two disorder-based groups in this population (namely, spinal cord and skeletal disorders). Understanding the FAT is important for supporting female players in these groups. This sport has physical demands and players are at risk of injuring bone. This study aimed to retrospectively investigate the FAT in female wheelchair basketball players on the Japanese national team and evaluate it from the perspective of spinal cord and skeletal disorders; this was intended to be a preliminary study for providing optimal support to para-athletes in the future.

## 2. Materials and Methods

### 2.1. Participants

We surveyed 13 female wheelchair basketball players from the Japanese national team of 2015. All players underwent a medical check-up including physical examination, radiological tests, and blood tests for participation in the international games. The study protocol was approved by the Human Ethics Review Committee of Ibaraki Prefectural University of Health Sciences (Approval Nos. 485 from 13 July 2012 and e45 from 10 May 2016), and was conducted according to the principles of the Declaration of Helsinki. This study was approved by the review board based on opt-out consent instead of the standard written consent form. All participants also provided verbal informed consent.

Demographic data were obtained through in-person interviews and self-reported questionnaires, and included data on age, height or arm span, weight, body mass index (BMI), and underlying disease. We classified underlying diseases into two groups, namely, spinal cord and skeletal system disorders. We also collected information on the players’ medical history including gynecological diseases, stress fractures, menstrual problems, and athletic history, and characteristics such as length of their athletic career and wheelchair usage conditions.

As previously stated, wheelchair basketball is classified into eight categories in accordance with pelvic stability [12,13]. We classified the participants into two groups based on pelvic stability as follows: class 1.0–2.5 (pelvic instability without sufficient trunk muscular strength) and class 3.0–4.5 (pelvic stability with sufficient trunk muscular strength). We classified wheelchair usage conditions into two groups, namely, daily-life wheelchair users and daily-life wheelchair non-users.

### 2.2. Laboratory Data

We evaluated the following laboratory data: white blood cell (WBC) count, red blood cell (RBC) count, and platelet (Plt) count, and levels of hemoglobin (Hb), total protein (TP), albumin (ALB), aspartate aminotransferase (AST), alanine aminotransferase (ALT), gamma-glutamyltranspeptidase (γGTP), alkaline phosphatase (ALP), creatine phosphokinase (CK), total cholesterol (TCHO), low-density lipoprotein cholesterol (LDL), triglycerides (TG), high-density lipoprotein (HDL), urea nitrogen (UN), uric acid (UA), creatinine (Cr), sodium ion (Na), potassium ion (K), chlorine ion (Cl), calcium ion (Ca^2+^), phosphorus (P), iron (Fe), and ferritin, which were necessary data to participate in the international game. In addition, we evaluated laboratory items regarding female hormone and the bone metabolism markers such as estradiol (E2), progesterone (PG), total P1NP (total procollagen type 1 N-terminal propeptide), and TRACP-5b (tartrate-resistant acid phosphatase 5b) to evaluate the FAT.

### 2.3. Nutritional and Body Composition Assessments

We used the food frequency questionnaire (FFQg) to assess nutritional status [17,18,19]; this assessed total energy, total protein, iron, and vitamin D intakes. 

We calculated the BMI using the arm span instead of height in participants who could not stand or had a deformity that would prevent adequate measurement of height. We investigated body composition using dual-energy X-ray absorptiometry (DEXA) [20] using the Delphi QDR4500 Bone Densitometer (Hologic, Marlborough, MA, USA) for evaluation of the players’ bone and muscle condition. For comparison with the values obtained for age-matched Japanese controls, we evaluated lean body mass (LBM), including both arms and legs [21], body fat percentage (BFP), BMD (total, both arms, and legs) and percentage of young adult mean BMD in healthy 40-year-old women (%YAM). We also calculated the index of EA as energy intake (kcal) minus exercise energy expenditure (kcal) divided by kilograms of LBM [1,22]. Energy expenditure was considered to be 1712 kcal/week based on the study by Abel et al. (2008) [23].

### 2.4. Statistical Analyses

Continuous variables are presented as means ± standard deviations. We determined the Spearman rank-correlation coefficient between EA and continuous data (age, athletic career, and blood test data). For nutritional analysis, we evaluated the correlations between the intake of total energy, protein, iron, calcium, and vitamin D and blood test data. In terms of body composition, we assessed the BMI and BFP in addition to LBM and BMD of both arms and legs, and used Welch’s t test to compare the total scores for each underlying disease, and wheelchair usage condition after using the Shapiro–Wilk test to evaluate normal distribution. In addition, we determined the Spearman rank-correlation coefficient between each body composition data (LBM, BMD, BFP, and BMI) and eight categorical classification (1.0–4.5). A *p* value < 0.05 was considered to indicate statistical significance. Analyses were performed using JMP 14.3.0 (SAS Institute Inc., Cary, NC, USA) software. The effect sizes were calculated using Cohen’s d. [24]; we also performed power analyses using JMP software.

## 3. Results

### 3.1. Demographic Data

The demographic data, athletic classification, and wheelchair usage conditions of the participants are shown in Table 1. Underlying diseases were grouped as spinal cord disorders (*n =* 8) and skeletal system disorders (*n =* 5). We also evaluated participants with menstrual disorders, including those with irregular menstrual cycles (*n =* 6), menstrual pain (*n =* 9), severe menstrual pain requiring painkillers or oral contraceptives (*n =* 3), and subjectively high menstrual flow (*n =* 3).

In addition, all spinal cord disorder players are low pointers (*n =* 8) and all skeletal players are high pointers (*n =* 5).

Table 2 shows the demographic data of each category, underlying diseases and wheelchair usage condition. The mean age of players with spinal cord disorders is younger and the mean athletic careers are longer; however, there are no statistical differences in ages and athletic careers in both groups.

### 3.2. Laboratory Data

The blood test measurement results are shown in Table 3. The iron level (63.4 ± 45.7 μg/dL) was higher than that at baseline (43 μg/dL); however, 5 players (38.5%) had iron levels below the baseline value. The mean serum ferritin (32.2 ± 29.0) was within the standard levels. One (7.7%) player had ferritin levels lower than the baseline value (4.0 µg/L), and six (3: spinal cord disorders, 3: skeletal disorders, 46.2%) had lower values than the cutoff level (20 µg/L) for able-bodied-athletes, as reported in previous studies [25,26].

Mean estradiol, progesterone, total P1NP, and TRACP-5b levels were 135.7 pg/mL, 7.1 ng/mL, 60.4 μg/L, and 300.7 mU/dL, respectively. Mean of all laboratory parameter values were within normal limits.

### 3.3. Nutritional and Body Composition Assessments

Table 4 shows the nutritional data obtained using the FFQg [17,18,19]. The total energy and protein intake almost met the standard requirements; however, the iron, calcium, and vitamin D intake levels were less than the required amounts.

Table 5 shows the body composition as evaluated using DEXA. In terms of the BMD, the %YAM was 109.2% ± 14.8%, and was comparable with the reference value for able-bodied women. Regarding LBM, the percentage of leg LBM was 67.8 ± 28.6%, which was lower than that of age-matched Japanese controls; however, the mass of the arms was 150.6 ± 16.9%, which was higher than that of controls [21]. The BFP was 27.2% ± 5.5%, which was higher than expected; the mean BMI was 21.7 ± 1.6 kg/m^2^, which was within normal limits.

Using total energy intake and LBM, we calculated the EA to be 41.4 ± 14.2 kcal/kg of the LBM; this was less than the 45 kcal/kg recommended for athletes [1,22].

### 3.4. Statistical Data Analyses for Each Category of the FAT

• Low EA

We found a significant correlation between EA and age (*ρ* = −0.63, *p* = 0.020). There was no correlation between BMD or LBM and total energy and intake of each nutrition.

• Menstrual Disorder

We also evaluated correlations among menstrual symptoms such as regular or irregular cycles, menstrual pain severity, menstrual flow, and blood data. The serum iron and hemoglobin levels correlated with the degree of reported subjective menstrual flow (ρ = −0.63, *p* = 0.019 and ρ = −0.56, *p* = 0.046, respectively). There was no statistically significance between the serum ferritin and the flow degree. We found no correlation between menstrual symptoms and female hormone data.

• BMD and LBM

We assessed the correlation between total, arm, or leg BMD and underlying diseases. Table 6 shows the BMD, %YAM, LBM, BFP, and BMI categorized based on underlying disease, and wheelchair usage conditions. 

Among spinal cord and skeletal disorders, no significant differences were found in the arm, total BMDs, and %YAM; however, a significant difference was found in leg BMD (*p* = 0.005, d = 2.84). Wheelchair usage conditions showed similar differences (*p* = 0.014, d = 1.98). We found significant differences in leg and total LBMs; however, the arm lean mass did not significantly differ in both categories. Regarding BFP, significant differences were found in terms of diseases, *p* = 0.014, d = 2.84, respectively; there were no differences in the BMI based on the presence of underlying diseases and wheelchair usage.

• Body composition and classification

We determined the Spearman rank-correlation coefficient between each body composition data (LBM, BMD, BFP, BMI) and the eight-category classification (1.0–4.5). We found a significant correlation between the eight-category classification and BMD in both legs (*ρ* = 0.73, *p* = 0.005), total LBM (*ρ* = 0.75, *p* = 0.003), LBM in the both legs (*ρ* = 0.70, *p* = 0.007), and BFP (*ρ* = −0.64, *p* = 0.017). 

## 4. Discussion

In this study, we retrospectively surveyed female wheelchair basketball players from the Japanese national team in terms of the FAT in order to more adequately support para-athletes, who have different characteristics from able-bodied athletes and need more monitoring of physical conditions.

Firstly, in those with menstrual disorders, the female hormone levels were within normal limits; however, certain athletes had subjective symptoms such as pain or an irregular cycle, and high blood flow. We did the feedback of the result for each player, and recommended that some players who had some menstrual trouble were seen by gynecologists. We observed significant correlations between quantities of blood flow and serum iron levels, and between the flow degree and hemoglobin levels. Although the mean iron levels were higher than that of baseline values, 5 players had lower serum iron levels than at baseline. The mean serum ferritin levels (32.2 ± 29.0) were within the standard levels. One (7.7%) player had lower ferritin levels than the baseline value (4.0 µg/L), and six (46.2%) had lower values than the cutoff level (20 µg/L) for able-bodied athletes [25,26].

Secondly, regarding nutritional status, iron intake levels were less than the recommended amounts. Our study participants showed lower EA (41.4 kcal/kg LBM) than that recommended for athletes (45 kcal/kg) [1,22]. EA and age showed a significant correlation, wherein younger athletes had better nutritional status. Nutritional intake may be related to living conditions or family structure. Thus, comprehensive support may be needed, particularly for older athletes who play while also working.

We conclude that these athletes require support, such as education and counselling, with respect to menstrual disorders and nutritional parameters including iron intake. Therefore, we recommend that the medical support staff for the wheelchair basketball team conduct some lectures on menstrual disorders every year.

Thirdly, we evaluated BMD using DEXA. We also evaluated body composition, namely LBM, BFP, and BMI. We used two categories of underlying diseases and wheelchair usage conditions. Among these categories, the leg BMD showed significant differences. Previous studies reported that patients with spinal cord disorders had a higher risk of osteoporosis [2,5]. Our study also found low BMD in the legs of players with spinal cord injuries, who were daily wheelchair users. However, the mean total BMD was appropriate for able-bodied women. Miyahara et al. compared the BMD between male wheelchair athletes and physically able athletes; they reported the arm BMD to be higher and the entire and leg BMDs to be lower in the former [27]. Goktepe et al. compared male paraplegic wheelchair athletes with male paraplegic controls and reported that the former had a higher BMD in the distal radius [28]. In previous studies, ball sports had the potential to maintain bone strength [29]. This suggests that playing wheelchair basketball may be beneficial for maintaining upper limb BMD. In this report, the mean total BMD in players with spinal cord injuries, daily wheelchair users, and low pointers was equivalent to that of age-matched controls owing to sufficient arm BMD. It also should be noted that the risk of traumatic or fragility fractures is particularly high among players with spinal cord injuries [5] or daily wheelchair use. Although the total BMD score was equivalent to that of the aged control group, bone insufficiency in the leg requires particular attention, especially in players with spinal cord injuries.

Regarding LBM, the mean arm lean mass was higher (150.6%) than that of the age-matched control population. Daily wheelchair users had higher arm lean mass than those who only used wheelchairs for playing basketball; however, there were no statistically significant differences. Wheelchair basketball and the protrusion of the wheelchair itself may increase arm muscle volume. DEXA has been shown to be an effective tool in the evaluation of wheelchair basketball players [20,30]. The assessment of body composition, LBM, BFP, and BMI showed that individuals with spinal cord disorders and frequent wheelchair use had considerable differences with respect to the arms and legs. In addition, a correlation was seen between the classification and leg BMD, total LBM, leg LBM, and BFP. This study showed that players with spinal cord injuries and daily wheelchair users, or players in the lower class, had lower leg BMD, total or leg LBM. BFP was higher in players with spinal cord injuries; however, there was no difference in BFP between daily wheelchair users and non-users. Understanding these differences in underlying disease, wheelchair usage conditions, and classifications is useful for nutritional support or muscle training. We should also share this information with nutritionists, trainers, and coaches.

This study has certain limitations. First, it had a cross-sectional design and small sample size. As the cohort comprised elite athletes from a group with disorders, the participants’ special characteristics precluded an increase in sample size. These findings should therefore be interpreted with care, and may be used merely as a reference for future support and prospective studies. Second, it is difficult to measure energy expenditure in real-time while players participate in wheelchair basketball. Studies with longer follow-up, including players’ physical conditions and circumstances of play are needed.

We comprehensively studied the impact of wheelchair basketball in terms of deep tissue injuries [14,15], shoulder pain [16], and sleep status [31,32] using a multi-support team. Players with each underlying disease had different body compositions and wheelchair usage conditions. Support for these individuals based on an understanding of their diseases and para-support, is imperative. In this study, we targeted female athletes; however, RED-S [2] is a concept that includes male athletes. This study may serve to improve support for para-athletes.

We plan to continue this survey to support wheelchair basketball players and use our findings to serve other para-sports. In addition, the findings from this study reiterate the importance of working in a multi-disciplinary support team, including medical doctors (physiatrists, orthopedists, and gynecologists), therapists, physical trainers, coaches, nutritionists, and other support staff, to offer some practical suggestions to assist the para-sport athletes and enhance their likelihood of impacting their performances as previously highlighted by other researchers in para-sport [33,34,35].

## 5. Conclusions

We investigated the FAT in female wheelchair basketball players in the Japanese national team. Certain athletes had gynecological issues. Players with high blood flow had lower serum Hb and Fe levels. The leg BMD of players with spinal disorders was significantly lower than that of those with skeletal disorders; however, the total BMD was comparable to that of the healthy population. The arm lean mass of the players was higher than that of able-bodied women. 

Wheelchair basketball has patients with two characters, spinal cord injured player, or skeletal system disorder players, or daily wheelchair users or not. We need to understand those two characteristics for adequate supports of para-athletes. 

Further research on the FAT is particularly necessary for optimizing health and sports performance in female para-athletes. These findings may help multi-disciplinary teams, including medical doctors, therapists, physical trainers, coaches, nutritionists, and other support staff, assist the para-athletes in performing better.

## Figures and Tables

**Table 1:** Participant characteristics.

	*n*	Mean ± SD or %
Age (years)	13	28.9 ± 8.1
Athletic career (years)	13	10.3 ± 6.3
BMI (kg/m^2^)	13	21.7 ± 1.6
Medical history		
Stress fractures	0	0%
Gynecological disease	2	15.4%
Menstrual problems		
Menstrual cycle disorder	6	46.2%
Menstrual pain	9	69.2%
Severe menstrual pain	3	23.1%
Subjective menstrual flow	3	23.1%
Underlying disease		
Spinal cord disorder	8	38.5%
Skeletal system disorder	5	61.5%
Classification		
Class 1.0–1.5	4	30.8%
Class 2.0–2.5	4	30.8%
Class 3.0–3.5	0	0%
Class 4.0–4.5	5	38.5%
Low pointer (Class 1.0–2.5)	8	61.5%
High pointer (Class 3.0–4.5)	5	38.5%
Wheelchair use		
Daily-life Wheelchair User	8	61.5%
Daily-life Wheelchair Non-user	5	38.5%

**Table 2:** Demographic data divided by underlying disease of wheelchair condition.

	Underlying Diseases				Wheelchair Usage Conditions			
	Spinal Disorder	Skeletal Disorder	*p* Value	Power	Daily Life	Only for Basketball	*p* Value	Power
N	8	5			8	5		
Age (year)	27.9 ± 9.8	30 ± 4.9	0.578	0.056	29.4 ± 10.4	28.2 ± 2.9	0.812	0.082
Career (year)	11.9 ± 7.1	7.9 ± 4.0	0.284	0.178	11.5 ± 7.4	8.5 ± 3.9	0.425	0.118
Class	2.1 ± 1.0	4.0 ± 0.9	** <0.001	1.000	1.9 ± 0.7	4.3 ± 0.3	** 0.005	0.894

** *p* < 0.01.

**Table 3:** Blood test data (*n* = 13).

	Standard Value	Mean ± SD
WBC (10^3^/μL)	3.04–8.54	7.1 ± 1.6
RBC (10^6^/μL)	3.78–4.99	4.5 ± 0.3
Hb (g/dL)	10.8–14.9	13.3 ± 0.7
Plt (10^3^/μL)	140–379	258.5 ± 46.2
TP (g/dL)	6.7–8.3	7.5 ± 0.5
ALB (g/dL)	3.9–4.9	4.5 ± 0.4
AST (IU/L)	8–38	18.9 ± 2.8
ALT (IU/L)	4–44	14.0 ± 3.6
γGTP (IU/L)	10–47	17.3 ± 11.0
ALP (IU/L)	104–338	208.7 ± 65.4
CK (IU/L)	43–165	98.6 ± 49.9
UN (mg/dL)	8–20	10.5 ± 3.9
Cr (mg/dL)	0.2–0.8	0.5 ± 0.2
UA (mg/dL)	3.6–7.0	3.7 ± 0.5
Na (mEq/L)	135–145	140.0 ± 0.9
K (mEq/L)	3.5–5.0	3.8 ± 0.4
Cl (mEq/L)	98–108	103.4 ± 1.5
Ca^2+^ (mEq/L)	8.5–10.2	9.7 ± 0.4
P (mEq/L)	2.5–4.5	3.8 ± 0.4
Fe (/dL)	43–172	63.4 ± 45.7
Ferritin (µg/L)	4.0–87.0	32.2 ± 29.0
E2 (pg/mL)	※	135.7 ± 61.6
P4 (ng/mL)	※※	7.1 ± 5.3
Total P1NP (μg/L)	16.8–70.1	60.4 ± 18.0
TRACP-5b (mU/dL)	120–420	300.7 ± 90.6

WBC: white blood cell, RBC: red blood cell, Hb: hemoglobin, Plt: platelet, TP: total protein, ALB: albumin, AST: aspartate aminotransferase, ALT: alanine aminotransferase, γGTP: gamma-glutamyltranspeptidase, ALP: alkaline phosphatase, CK: creatine phosphokinase, UN: urea nitrogen, Cr: creatinine, UA: uric acid, Na: sodium ion, K: potassium ion, Cl: chlorine ion, Ca^2+^: calcium ion, P: phosphorus, Fe: iron, E2: estradiol, P4: progesterone, total P1NP: total procollagen type 1 N-terminal propeptide, TRACP-5b: tartrate-resistant acid phosphatase 5b. ※ follicular phase: 19–226, ovulation phase: 49–487, luteal phase: 78–252. ※※ follicular phase: −0.4, ovulation phase: −3.7, luteal phase: 8.5–21.9.

**Table 4:** Nutritional data.

	Intake	Percentage of Age-Specific Intake Requirements
Total energy (kcal)	1636.1 ± 439.5	99.8 ± 28.0
Protein (g)	57.5 ± 18.9	134.4 ± 44.3
Iron (mg)	6.4 ± 2.5	59.8 ± 24.3
Calcium (mg)	462.9 ± 240.1	71.2 ± 36.9
Vitamin D (IU)	4.8 ± 3.1	81.4 ± 52.3

**Table 5:** Body composition.

		Mean ± SD
BMD (g/cm^2^)	Both arms	0.79 ± 0.07
	Both legs	1.07 ± 0.32
	Total	1.20 ± 0.16
	%YAM	109.2 ± 14.8
LBM (kg)	Both arms	4.97 ± 0.56
	Both legs	9.35 ± 3.83
	Total	34.85 ± 6.55
BFP		27.23 ± 5.52
BMI (kg/m^2^)		21.71 ± 1.59

**Table 6:** Body composition categorized by underlying disease, and wheelchair usage.

Underlying Diseases/Classification						
		Spinal Disorders Low Pointers	Skeletal Disorders High Pointers	*p* Value	Effect Size d	Power
		Mean ± SD	Mean ± SD			
BMD (g/cm^2^)	Both arms	0.79 ± 0.09	0.78 ± 0.03	0.462	0.22	0.064
	Both legs	0.87 ± 0.07	1.40 ± 0.08	** 0.005	2.84	0.995
	Total	1.15 ± 0.18	1.28 ± 0.11	0.125	0.85	0.273
%YAM		104.6 ± 16.0	116.5 ± 9.8	0.125	0.85	0.273
LBM (kg)	Both arms	4928.2 ± 626.5	5039.6 ± 488.2	0.723	0.2	0.061
	Both legs	13538.1 ± 617.7	6733.6 ± 3140.9	** <0.001	3.89	1.000
	Total	30103.3 ± 2480.8	42441.3 ± 809.9	** <0.001	6.05	1.000
BFP		30.1 ± 3.9	22.7 ± 4.7	* 0.021	1.76	0.800
BMI (kg/m^2^)		21.9 ± 1.6	21.5 ± 1.7	0.702	0.23	0.066
wheelchair usage conditions						
		Daily life	Only for basketball	*p* value	Effect Size d	Power
		Mean ± SD	Mean ± SD			
BMD (g/cm^2^)	Both arms	0.80 ± 0.09	0.77 ± 0.02	0.473	0.34	0.086
	Both legs	0.89 ± 0.21	1.35 ± 0.27	* 0.014	1.98	0.884
	Total	1.18 ± 0.18	1.24 ± 0.13	0.476	0.39	0.096
%YAM		106.9 ± 16.6	112.8 ± 12.0	0.476	0.39	0.096
LBM (kg)	Both arms	5122.0 ± 513.6	4726.4 ± 594.2	0.256	0.73	0.214
	Both legs	7413.8 ± 3328.7	12449.7 ± 2262.9	** 0.008	1.69	0.768
	Total	31592.0 ± 5463.4	40059.3 ± 4597.7	* 0.014	1.64	0.745
BFP		28.4 ± 6.0	25.4 ± 4.6	0.338	0.54	0.138
BMI (kg/m^2^)		22.0 ± 1.6	21.2 ± 1.6	0.402	0.50	0.126

* *p* < 0.05, ** *p* < 0.01.
